# Recent magmatism drives hydrocarbon generation in north-east Java, Indonesia

**DOI:** 10.1038/s41598-020-58567-6

**Published:** 2020-02-04

**Authors:** Alexandra Zaputlyaeva, Adriano Mazzini, Martin Blumenberg, Georg Scheeder, Wolfram Michael Kürschner, Jolanta Kus, Morgan Thomas Jones, Joost Frieling

**Affiliations:** 1 Centre for Earth Evolution and Dynamics (CEED), University of Oslo, Oslo, Norway; 2 Federal Institute for Geosciences and Natural Resources (BGR), Hannover, Germany; 3 Department of Geosciences, University of Oslo, Oslo, Norway; 4 Department of Earth Sciences, Utrecht University Utrecht, Netherlands

**Keywords:** Geochemistry, Natural hazards, Geology

## Abstract

Conventional studies of petroleum basins associate oil generation with the gradual burial of organic-rich sediments. These classical models rely on the interplay between pressure, temperature, and the time required for organic matter transformation to oil and gas. These processes usually occur over geological timescales, but may be accelerated by rapid reactions when carbon-rich sediments are exposed to migrating magmatic fluids. The spectacular Lusi eruption (north-east Java, Indonesia) is the surface expression of the present-day deep interaction between volcanic and sedimentary domains. Here we report the ongoing generation of large amounts of hydrocarbons induced by a recent magmatic intrusion from the neighbouring Arjuno-Welirang volcanic complex. We have investigated a unique suite of oil and clast samples, and developed a detailed conceptual model for the complex hydrocarbon migration history in this part of the basin by integrating multidisciplinary techniques. Our results show that palynology, organic petrology, and chlorite microthermometry are the most sensitive geothermometers for basins affected by recent magmatic activity. These findings further our understanding of the driving mechanisms fueling the world’s largest active mud eruption and provide a unique dataset to investigate modern hydrocarbon generation processes.

## Introduction

**Figure 1:**
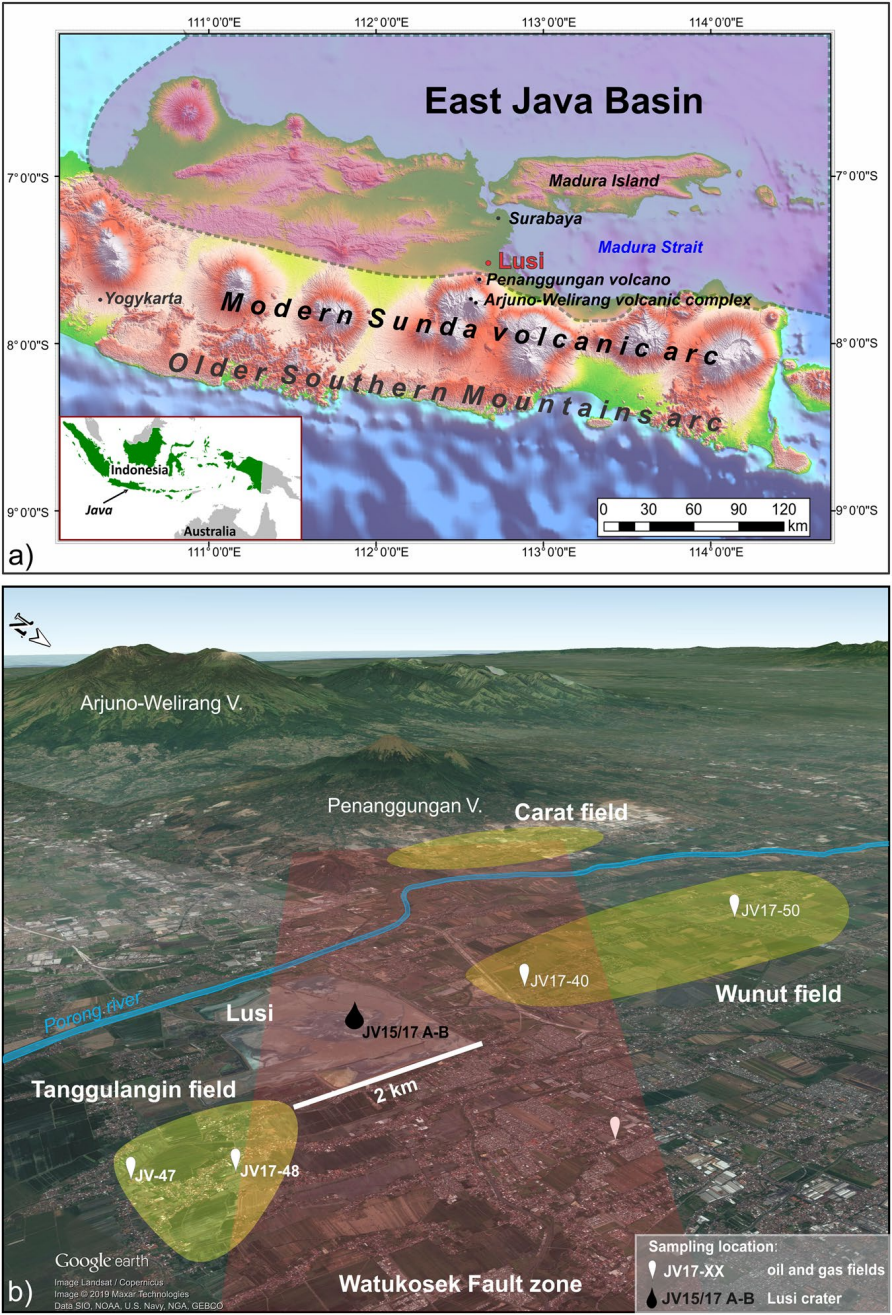
(**a**) Topography of the central and eastern Java, showing the location of Lusi mud eruption. The East Java sedimentary basin is highlighted by the purple-shaded area. Map of Indonesia in the inset. Topographic data is from the USGS SRTM (Shuttle Radar Topography Mission 1 Arc-Second Global, Source: Global Land Cover Facility). Map created using ArcGIS v10.5 (www.arcgis.com). (**b**) 3D Google Earth view of the study area, indicating the locations of the sampled Wunut and Tanggulangin hydrocarbon fields and Lusi crater. The red shaded area indicates the Watukosek fault system. Map data: Google, Image Landsat/Copernicus, Maxar technologies, SIO, NOAA, U.S. Navy, NGA, GEBCO.

Hydrocarbons (HCs) stored in the sedimentary basins are predominantly of biotic origin, i.e. they are derived through the alteration of buried organic matter (OM)^1^. The deposited OM experiences various alteration stages during diagenesis, catagenesis and metacatagenesis leading to HC generation. Some HCs (mostly methane) form at relatively modest temperatures (below 60–80 °C) due to microbial activity. In contrast, a larger variety of HCs (especially oil) are generated at higher temperatures (>60 °C) during thermocracking processes of kerogens^2–5^. Oil generation typically occurs at significant burial depths (commonly 1.5–4 km, depending on the geothermal gradient and OM type) over timescales of thousands to millions of years. However, regardless the burial history and temperature conditions, rapid oil generation can be triggered if the source rocks are exposed to anomalously high heat induced by magmatic/hydrothermal activity. This phenomenon has been documented at several localities worldwide including the Guaymas Basin, Escanaba Trough, Lake Tanganyika, Neuquén Basin, Rockall Trough, Vøring-Møre basins, Salton Sea, and Faroe-Shetland basins^6–9^. The overpressure produced by thermo-metamorphic reactions of organic matter exposed to high temperatures may lead to the formation of piercements that reach the surface, forming the so- called sediment-hosted geothermal systems (SHGSs)^10^. The largest documented SHGS on Earth is the ongoing Lusi mud eruption (named after LUmpur, “mud” in Indonesian, and SIdoarjo, the Local Regency), active since May 2006 in the East Java sedimentary basin, Indonesia (Fig. 1a). The study area is located just 10 km from the active Sunda volcanic arc. Several studies, including gas and water geochemical surveys and ambient noise tomography, show that the volcanic complex and Lusi plumbing system are connected through a fault system (Watukosek fault system) at a depth of ~4.5 km^11–16^.

Lusi is surrounded by three hydrocarbon fields: Wunut, Tanggulangin, and Carat (Fig. 1b). These fields contain oil and gas accumulations within the shallow (200–1000 m depth) volcaniclastic reservoirs of the Pleistocene Pucangan Formation (Fm.). The Tanggulangin and Wunut fields are currently producing gas, and the oil production has ceased. Lusi is continuously erupting water, gas, rock clasts, mud and oil. The sources of water, gas and mud are relatively well constrained^12–17^. In order to distinguish the source of oil and identify the potential migration pathways, we present new geochemical, palynological, and petrographical data acquired from oil films and rocks clasts erupted at the Lusi vent and compare them with those obtained from oil samples from the neighbouring HC fields.

The East Java sedimentary basin is located on the south-eastern margin of the Sunda plate, in the back-arc of the Sunda volcanic arc that has been active since the Miocene (ca. 12–10 Ma). The older Southern Mountain arc, located 50 km further south, was active between ca. 45–20 Ma and was also formed due to northward-directed subduction of the Indian*-*Australian Plate under the Sunda plate^18,19^. The Penanggungan and Arjuno-Welirang Holocene volcanoes are located 10 and 25 km, respectively, to the south-west of the Lusi mud eruption site (Fig. 1).

**Figure 2:**
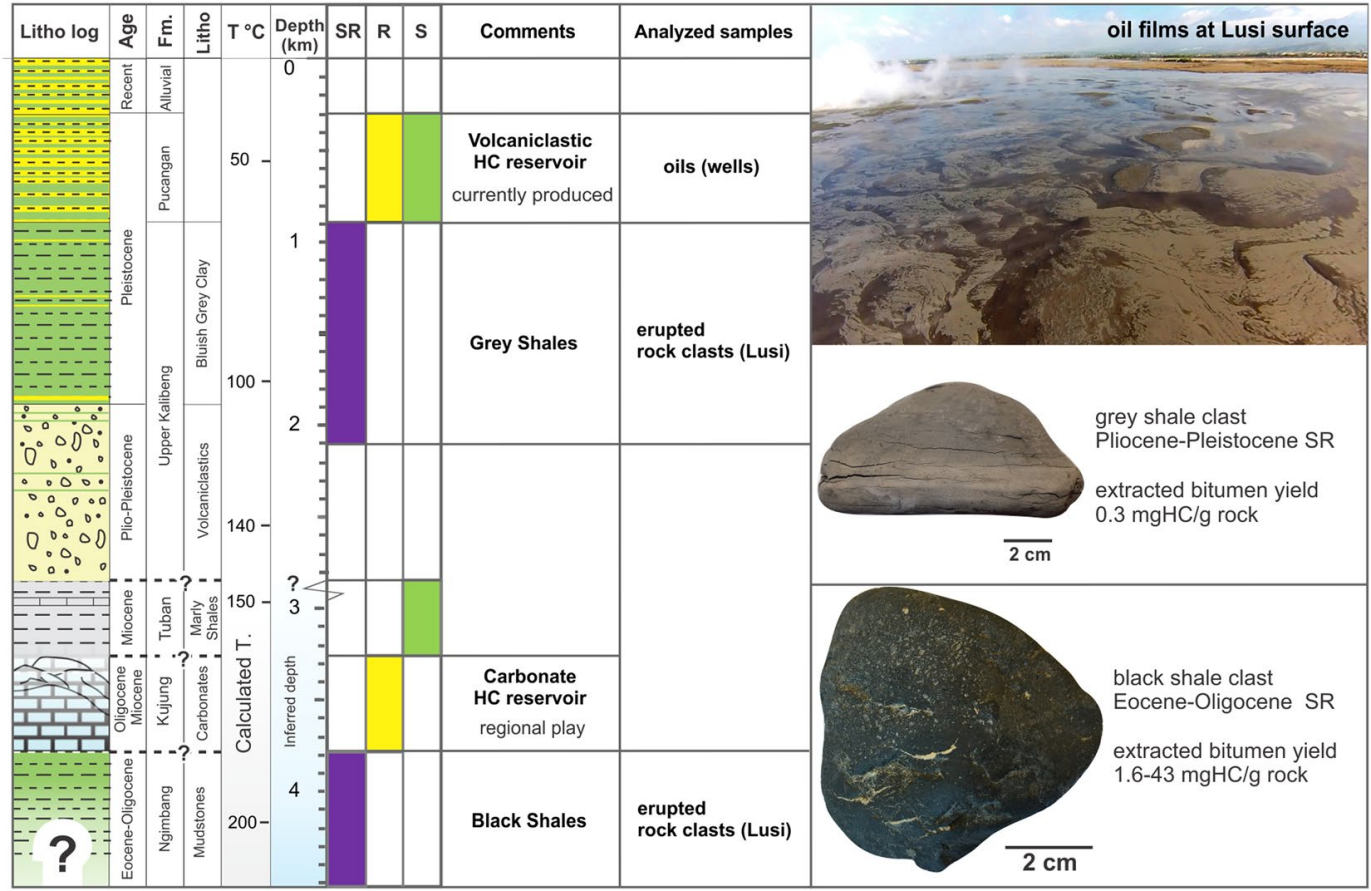
Petroleum systems of the southern part of the East Java petroleum basin, highlighting the main HC source rocks (SR), reservoirs (R), seals (S) and examples of the analysed samples with corresponding formation origin. Temperature gradient was measured in the well BJP-1 (located at the Lusi site) and inferred below 2.8 km^20^. Lithostratigraphy log is from Samankassou *et al*.^17^.

In the study area the basin consists of a >5 km thick sedimentary section that overlies Pre-Cenozoic basement (Fig. 2). The lithostratigraphy of the region is constrained by the drilled boreholes and seismic surveys acquired in 1990’s-2000’s^13,17,20–22^. The sedimentary section contains (from bottom to top): Middle Eocene-Lower Oligocene organic-rich black shales of the Ngimbang Fm. (>3800 m), Upper Oligocene-Lower Miocene carbonates of the Kujung Fm. (from ~3800 to ~3250 m), Lower-Upper Miocene marls and shales of the Tuban Fm. (from ~3250 to ~2830 m), Upper Pliocene-Pleistocene Upper Kalibeng Fm. containing tight volcanic and volcaniclastic units in the lower part (~2830 to 1870 m) and bluish grey shales and marls in the upper part (1870–900 m), Pleistocene volcaniclastic shales and sands of the Pucangan Fm. (900–290 m), and recent alluvial sediments (290–0 m). This part of the basin is characterized by high sedimentation rates (0.7 km/Myr) since Late Pliocene, which caused the rapid burial and preservation of the semi-lithified deposits.

The East Java sedimentary basin is a petroleum-rich region, with a total estimated reserves of 1830 Million Barrels of Oil Equivalent^23^. The hydrocarbon accumulations in the basin were discovered in Pleistocene volcaniclastic reservoirs of the Pucangan Fm., Miocene sands of the Ngrayong Fm. and Woncolo Fm., Upper Oligocene-Lower Miocene carbonates of the Kujung Fm., and the carbonates and sands of the Ngimbang Fm.^23,24^. The organic-rich shales are confined to the Middle Eocene-Lower Oligocene Ngimbang Fm., Miocene Tuban Fm., Upper Pliocene-Pleistocene Upper Kalibeng Fms. The major HC source rock is the Ngimbang Fm., consisting of organic-rich shales, coals and coaly shales^22,25^.

In the study area, the producing reservoir intervals are confined to the Pucangan Fm., 200–1000 m depth, consisting of fine-grained lithologies (up to 80% of net shales) and interbedded with 3–50 m thick layers of sandstones^26^ (Fig. 2). The intercalating shales seal the HC accumulations. The gas accumulations have an oil leg in the lower intervals of the Pucangan Fm.

## Results

The dataset was gathered from a suite of samples collected during yearly sampling campaigns conducted in the study area since the beginning of the Lusi activity. Lithoclasts erupted at the crater site include carbonates, lahar, grey shales, and black shales. Among those, specimens of potential source rocks (grey and black shales) were selected for this study (30 rock clasts). In addition, 4 oil film samples collected from the Lusi crater and 4 oils samples from the Wunut and Tanggulangin oil and gas fields were analysed.

### Oils

**Figure 3:**
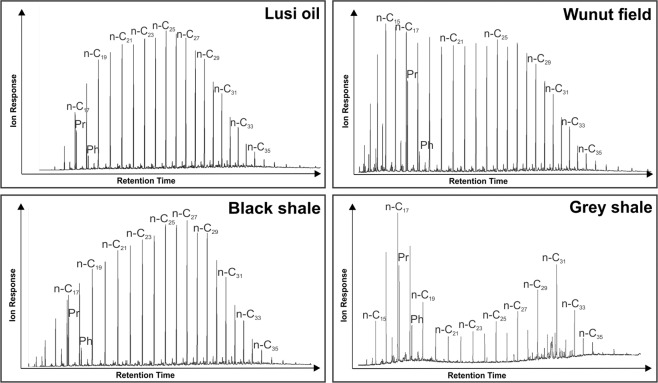
Gas chromatography results of the aliphatic fractions of the Lusi oil films, oil from the Wunut field, black and grey shale bitumen extracts. Normal and *iso*-alkane distribution of the Lusi oil films, oil from the Wunut HC field and bitumen extract of the black shales (Ngimbang Fm.) share similar distribution patterns, indicating that both oils originate from the clasts of the Ngimbang Fm. Low *n*-C_13_–C_17_ peaks in the black shale bitumen extracts and Lusi oil are due to the loss of light oil fraction in the Lusi vent, that was subjected to the temperatures greater than 100 °C. Alkane distribution in the bitumen extracts of the grey shales indicates that OM is immature and differs from that of black shales.

Organic geochemical analyses of the oil films collected from the Lusi crater (Supplementary Table S1) reveal that they are composed of 53–64% of saturated HCs, 13–15% of aromatic HCs, and 21–34% of polar compounds (i.e. resins and asphaltens). *n*-Alkane distributions demonstrate the predominance in high-molecular-weight zone (*n-*C_23_ − *n-*C_27_) (Fig. 3), Pristane/Phytane (Pr/Ph) ratios vary from 2.6 to 3.9. Carbon preference index ranges from 1.02 to 1.03 (CPI = (*n-*C_23_ + *n-*C_25_ + *n-*C_27_) + (*n-*C_25_ + *n-*C_27_ + *n-*C_29_)/2 × (*n-*C_24_ + *n-*C_26_ + *n-*C_28_) (Fig. 3). The oleanane index, indicating contributions to the organic matter of the source rock from certain angiosperms^27^, varies from 0.18 to 0.20, and 2- methylhopanoid index ranges from 0.05 to 0.06, and 3-methylhopanoid index in the samples is 0.03. The methylphenanthrene index [MPI-1 =1.5 *(3- + 2-MPhenanthrene)/(Phenanthrene +1- + 9-MPhenanthrene)^28^] ranges from 0.55 to 0.62, methylphenanthrene ratio (MPR = 2-MPhenanthrene/1-MPhenanthrene)^29^ equals to 1.18.

The oil sample from the Wunut field (JV17–40) is composed of 81% saturated HCs, 14% aromatic HCs, and 5% polar compounds. Normal and *iso*-alkane distributions are bimodal, with a predominance in lower molecular-weight zone (*n-*C_15_) and higher molecular-weight zone (*n-*C_23_ − *n-*C_27_) (Fig. 3). This sample was not affected by biodegradation processes. In contrast, oils from the other wells feature evidence of biodegradation occurring in the reservoirs, resulting in the reduction of the *n*-alkanes and increase of the *iso*-alkanes. Pr/Ph ratios of the 4 analysed oil samples vary from 4.1 to 4.34, while CPIs range from 0.94 to 1.06. The oleanane index ranges from 0.17 to 0.24, and 2- and 3-methylhopanoid indices vary from 0.056 to 0.059 and from 0.031 to 0.033, respectively. MPI-1 and MPR vary from 0.73 to 0.82 and from 1.19 to 1.22, respectively (Supplementary Table S1).

### Rock clasts

Rock clast samples were divided into 2 groups based on lithology: ***grey shales (GS) and black shales (BS)***. GS group contains light grey-coloured shales and marls with no obvious HC odour, often laminated, and poorly lithified. The BS group consists of black-coloured clasts, well-lithified, often laminated and with a strong HC odour.

**Figure 4:**
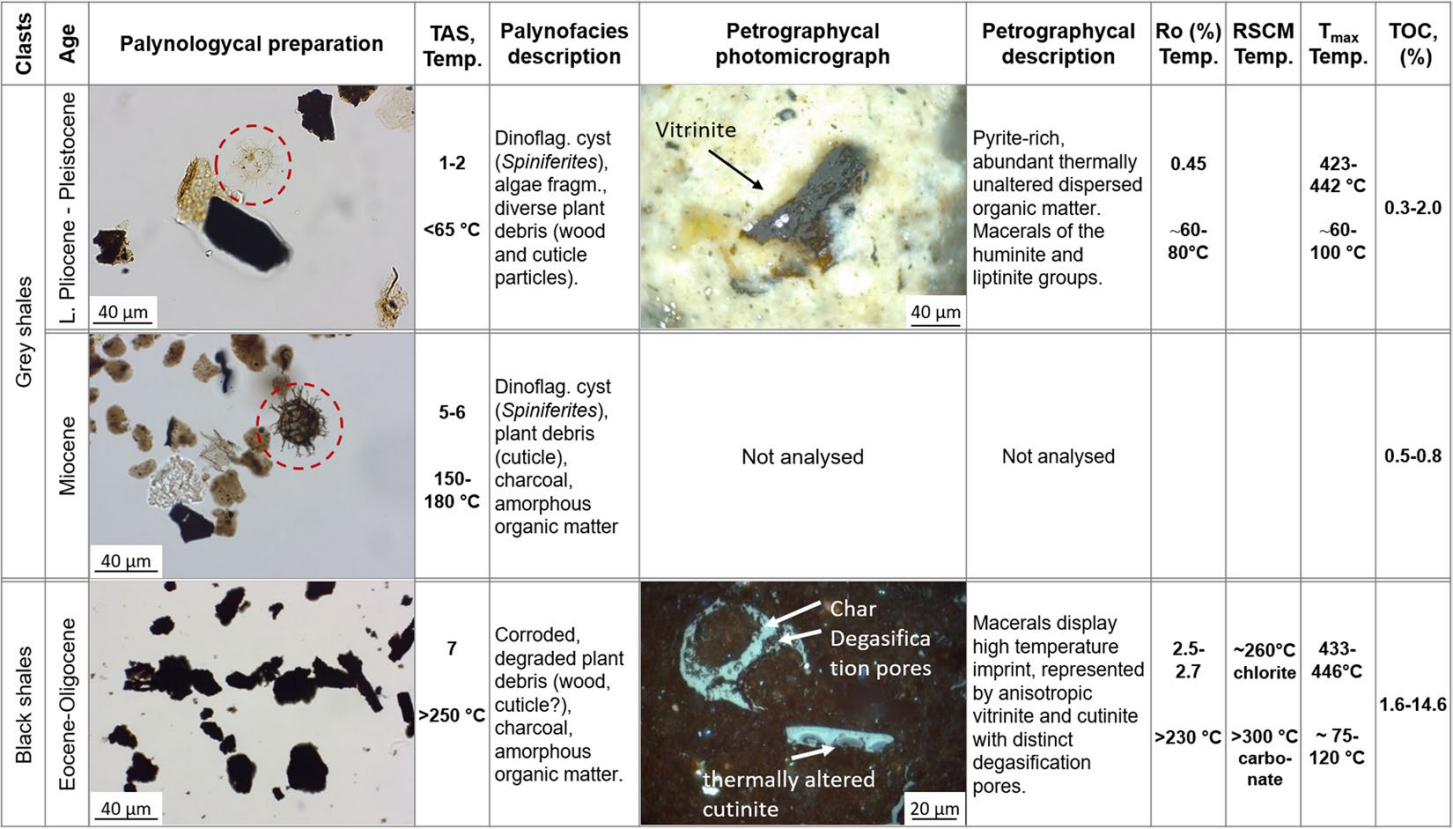
Summary chart of the identified lithostratigraphy groups with main observations and analysed thermal maturity parameters. TAS - Thermal Alteration Scale^33^, Temp. – estimated temperature, Ro – vitrinite reflectance measurements, T_max_ – Rock-Eval parameter characterizing the maturity of the samples, RSCM – Raman spectroscopy (from Malvoisin, *et al*.^13^), TOC – Total Organic Carbon. The shallow Upper Pliocene-Pleistocene grey shales (Up. Kalibeng Fm.) have lower maturity than the Eocene-Oligocene black shales (Ngimbang Fm.). Although black shales feature high temperature imprints (>300 °C), they consistently show high organic carbon content and moderately low T_max_ parameters, suggesting the temperatures lower than 120 °C. This discrepancy is ascribed to the migration of magmatic fluids within the Ngimbang Fm. that occurred only recently (possibly Holocene) and enhanced OM maturation.

*Palynological analysis* revealed that the GS clasts can be assigned to stratigraphical units ranging from Miocene to Late Pliocene-Pleistocene, corresponding to the Tuban and Upper Kalibeng Fms., respectively (Fig. 4, Supplementary Table S2). The shortage of quantitative palynological data and the long stratigraphic range of most palynomorphs do not allow a more detailed biostratigraphic correlation to regional palynological zonation schemes^30–32^. However, GS samples contain both marine and terrestrial palynomophs, including the long-ranging dinoflagellate cysts (dinocysts) species *Lingulodinium machaerophorum*, *Spiniferites* spp., *Operculodinium* spp. and diverse spores and angiosperm pollen, including mangrove palm pollen (*Nypa*). The occurrence of dinocysts with a more limited range, such as *O*. *piaseckii* and *Dapsilidinium* sp. indicates a Miocene and pre-Pleistocene age for GS samples JV14B-02 and JV17–01–40, respectively (Supplementary Table S2). Additional constraints regarding the age of the samples can be provided using the colour of the palynomorphs that show 2 distinctive groups: a) yellow-orange, indicating the lowest thermal maturity, TAS (Thermal Alteration Scale^33^) = 1–2, < 65 °C, likely Late Pliocene-Pleistocene; and b) dark brown colours indicating a higher thermal maturity (TAS = 5–6, 150–180 °C), likely of Miocene age.

Palynological residue of the BS samples only contains highly thermally-altered and degraded/oxidized organic particles of black colour; identifiable palynomophs were lacking (TAS = 7, >250 °C, Fig. 4, Supplementary Table S2). Bleaching of the organic residue revealed a terrestrial palynofacies with charcoals, leaf cuticles, highly degraded amorphous OM, some highly corroded spores, and remains of fresh water algae. The high thermal maturity and the extensive degree of alteration observed in these remains hampered an age assignment using biostratigraphic events. However, the palynofacies of the BS samples consistently indicate a terrestrial, fluvial-lacustrine depositional environment, which suggests that the BS lithoclasts originate from the lower terrestrial units of the Eocene–Oligocene Ngimbang Fm. Previous studies showed that the lower part of Ngimbang Fm. was deposited in a terrestrial to coastal marine setting with sands, coals and lacustrine shales, overlain by marine shales and limestones in the upper part^31,34^. While younger lithostratigraphic units (the Upper Kalibeng and Tuban Fms) were deposited predominantly in a marine environment. This is supported by the regular occurrences of open marine dinocyst genera, such as *Spiniferites* and *Operculodinium*^35^ in the GS samples, which are correlated to the Upper Kalibeng and Tuban Fms.

**Figure 5:**
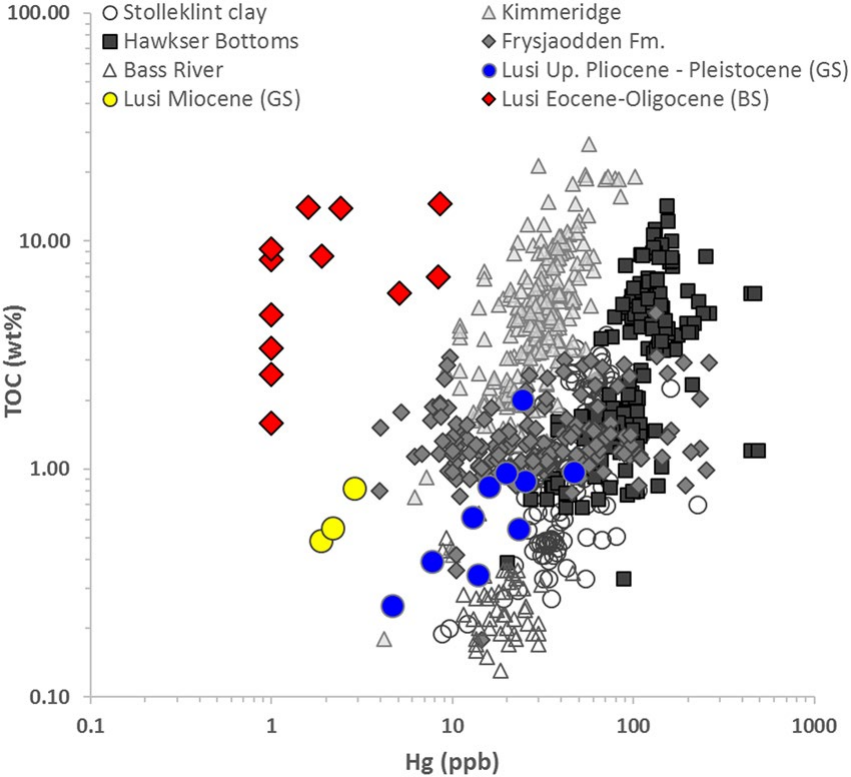
The distribution of mercury and TOC concentrations in the black shales (BS) and grey shales (GS) samples from the Lusi eruption site. The grey data points correspond to the shale sections from published datasets from the Paleocene-Eocene Stolleklint clay in Fur island (Denmark), Paleocene-Eocene Frysjaodden Fm. (Svalbard), Paleocene-Eocene Bass River locality (USA), Upper Jurassic Kimmeridge clay (UK), and Low Jurassic Hawkser Bottoms (UK)^59,60^ showing the known range of Hg/TOC values in clays and shales. Note that Hg concentrations <1 ppb are below the instrumental detection limit and are here given a value of 1 for illustration purposes.

*Mercury concentrations* in Upper Pliocene-Pleistocene GS samples range from 5 to 47 ppb (mean 20.9 ppb, Fig. 5), in Miocene GS samples from 2 to 3 ppb (mean 2.3 ppb), in the Eocene–Oligocene BS samples is from 1 to 9 ppb (mean 2.8 ppb). There is a strong positive correlation between Hg and TOC in modern sediments as OM is usually the dominant depositional pathway for Hg into sediments^36^. Mercury concentrations are therefore often normalised to TOC concentrations to account for variations in OM deposition^37^. After normalisation, there is a distinctive and consistent difference between the BS clasts of the Eocene-Oligocene Ngimbang Fm. (Hg/TOC = 0.1 to 1.2 ppb/wt.%) and both the Miocene Tuban Fm. (Hg/TOC = 3.5 to 4 ppb/wt.%) and Upper Pliocene-Pleistocene Upper Kaliberg Fm. (Hg/TOC = 12 to 49 ppb/wt.%) GS clasts.

#### Organic petrography

The investigated Upper Pliocene-Pleistocene GS sample is a pyrite-rich marly shale with abundant thermally unaltered dispersed organic matter (Fig. 4). Macerals of the huminite and liptinite maceral groups were observed, among others ulminite, gelinite, as well as lamalginite, telalginite, sporinite, cutinite, liptodetrinite, and interodetrinite. Mean random vitrinite reflectance measurements show Ro = 0.45%. In contrast, the investigated Eocene–Oligocene BS samples are identified as silty shales with some inclusions of coarse crystalline quartz or dolomite. The groundmass encloses fine-pored structures characterised by pale inner reflections of unknown origin, possibly of a relict character. The encountered macerals display clear high temperature imprint and are represented by anisotropic vitrinite and cutinite with distinct degasification pores. The secondary particles embrace fly ash-like particles and pyrolitic carbons with characteristic spherulitic domains. Thermal maturity analyses based on the random vitrinite reflectance method show Ro = 2.47–2.69%.

#### Organic geochemistry

**Figure 6:**
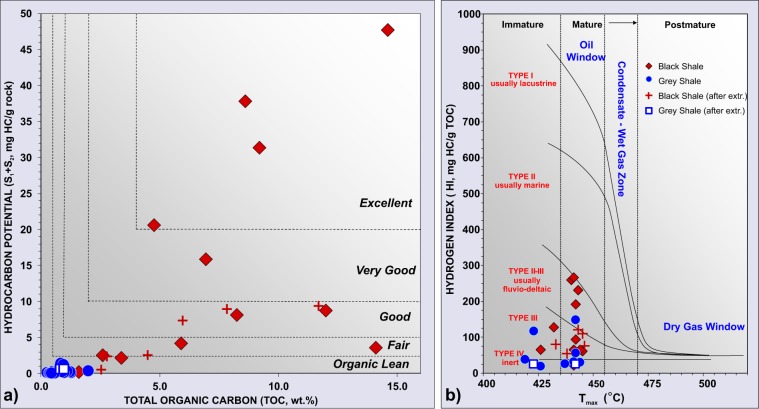
(**a**) Source rock characterization based on Rock-Eval parameters. TOC - Total Organic Carbon and S_1_ + S_2_ - hydrocarbon generative potential. Black shales have fair to excellent HC potential, while grey shales could be treated as organic lean (classification is based on Peters *et al*.^4^). (**b**) Maturity of the HC source rocks, suggesting that both grey and black shales fall within the oil window zone.

The Miocene-Pleistocene GS samples have low to moderate OM content (TOC from 0.3 to 2 wt.%), low generative potential (S_1_ peaks vary from 0.01 to 0.3 mg HC/g rock, S_2_ from 0.04 to 1.2 mg HC/g rock) and extracted bitumen amounts ranging between 0.26 to 0.28 mg/g rock (Fig. 6, Supplementary Table S3). The T_max_ is 419–444 °C. The extracted bitumens contain 7–15% saturated HCs, 24–34% aromatic HCs, 59–61% polar compounds. C_27–29_ sterane distributions indicate that the OM is of II-III type, fluvio-deltaic – shallow marine origin, which is in line with HI values up to 148 mg HC/g TOC (Fig. 6). Gas chromatography of the aliphatic fractions shows bimodal peak distributions reaching maximums in the lowmolecular-weight zone (*n-*C_17_) and high molecular-weight zone (*n-*C_31_) (Fig. 3). The Pr/Ph ratios are 0.5–2.5, with CPI values between 1.4–1.6. The oleanane indices range from 0.32 to 0.47, and 2- and 3-methylhopanoid indices vary from 0.025 to 0.026 and from 0.023 to 0.026, respectively. MPI-1 ranges from 0.41 to 0.56, MPR varies from 1.06 to 1.17 (Supplementary Table S1).

All Eocene–Oligocene BS clasts show distinct characteristics. The rocks have high OM contents (TOC from 1.6 to 14.6 wt.%, mean 7.7 wt.%), high generative potential (S_1_ from 0.1 to 19.8, mean 5.3 mg HC/g rock, S_2_ from 0.2 to 27.9 mg HC/g rock, mean 10 mg HC/g rock) and high extractable bitumen amounts (from 1.6 to 43.4 mg/g rock, mean 24.6 mg/g rock) (Fig. 6, Supplementary Table S3). The bitumens contain 53–91% (average 82%) saturated HCs, 7–24% aromatic HCs, 0.01–22% polar compounds. The OM is a II-III kerogen type with HI up to 267 mg HC/g TOC, which points to a fluvio-deltaic origin. The T_max_ is 433–446 °C and aliphatic fractions show unimodal peak distribution with maxima in the higher molecular-weight zone (*n-*C_25_ − *n-*C_29_) (Fig. 3). The Pr/Ph ratios are 2.6–3.7, with CPI values between 1.02–1.04. The oleanane index varies from 0.16 to 0.23, and 2- and 3-methylhopanoid indices vary from 0.051 to 0.060 and from 0.033 to 0.039, respectively. MPI-1 ranges from 0.63 to 0.9, MPR varies from 1.03 to 1.57 (Supplementary Table S1).

## Discussion

**Figure 7:**
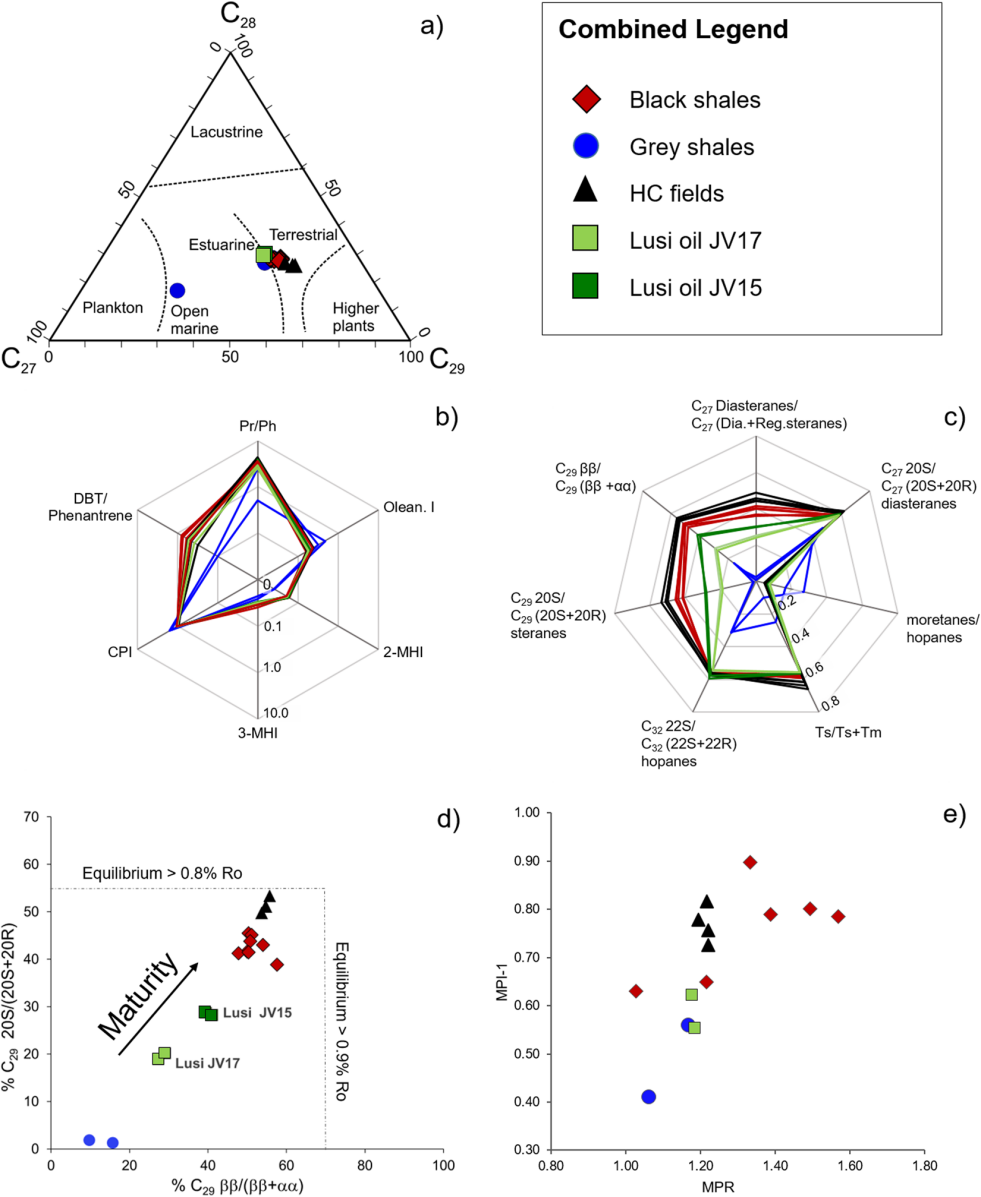
Biomarker and aromatic compounds distribution of the oil films from the Lusi, HC fields and bitumen extracts of the black and grey shale rock clasts. (**a**) Ternary diagram of the C_27_, C_28_ and C_29_ sterane distribution, indicating fluvio-deltaic – shallow marine depositional settings for most of the samples. (**b**) Star diagram of the major source-related biomarkers and aromatic compounds, suggesting that all Lusi oil films and oils in the HC fields are sourced by the black shales of the Ngimbang Formation (Eocene-Oligocene). 2-MHI and 3-MHI correspond to 2- and 3-methylhopanoid indeces, Olean. I - oleanane index, DBT - dibenzothiophene. (**c**) Star diagram of maturity-related biomarkers shows that grey shales have the lowest maturity, while the oils from the hydrocarbon fields have the highest maturity; (**d**) C_29_ sterane isomer distribution indicates that Lusi oil films and oils from the HC fields form different clusters, underscoring recent HC generation for the oil erupted at Lusi; e) methylphenanthrene index (MPI-1 = 1.5*(2-MP + 3-MP)/(P + 1-MP + 9-MP) and methylphenanthrene ratio (MPR = 2-MP/1-MP) suggest the lowest maturity for the Lusi oil, but the highest for the bitumen extracts of the BS samples.

High temperatures (~100 °C) at the surface of the Lusi mud vent may have caused partial leaching of the light oil fraction (particularly C_7_–C_16_ compounds) from the oil films and bitumens in the rock clasts. Nevertheless, oil-films from Lusi and oils from the HC fields share similar biomarker compositions with the bitumen extracts from the BS rock samples, including: a) unimodal *normal* and *iso*-alkane distribution (CPI ranging from 0.94 to 1.06); b) regular C_27_, C_28_, C_29_ sterane homologues distributions; c) oleanane index; d) 2- and 3-methylhopanoid indices; e) as well non-biomarker dibenzothiophene/phenanthrene ratio (DBT/Phen) (Figs. 3 and 7a,b, Supplementary Table S1 and Fig. S4). These biomarkers and aromatic compounds ratio are commonly used for source-oil and oil-oil correlations^27,38–40^. Extractable organic matter from the Miocene-Pleistocene GS samples have remarkably different characteristics, which include: a) bimodal alkane distribution, with predominance of the odd over even alkanes (CPI ranges from 1.4 to 1.6); b) higher oleanane index than in the oils and BS bitumen extracts; c) lower 2- and 3-methylhopanoid indices; d) lower DBT/Phen (Figs. 3 and 7a,b, Supplementary Table S1 and Fig. S4). Rock-Eval analysis reveals that GS samples have poor hydrocarbon generative potential^4^, implying that these formations can generally only negligibly contribute to petroleum systems in this part of the basin (Fig. 6a). In contrast, BS clasts have high OM content, and the hydrocarbon generative potential for most of the samples is good to excellent. These observations suggest that all the oils, both vented at Lusi and trapped in the HC reservoirs, share a common origin pointing to the Eocene–Oligocene BS clasts of the Ngimbang Fm. as the major HC source rock.

The high occurrences of oleanane, as well as high Pr/Ph ratios, DBT/Phen ratio lower than 1 (Fig. 7a,b, Supplementary Table S1), combined with results from palynology, clearly indicate a dominant terrestrial source of the organic matter of the HC source rock. These characteristics along with the lithology and the high OM content match the geochemical results obtained from the wells penetrating the Ngimbang Fm. source rock^25^, the dominant source rock in the East Java Basin^22,23^.

The thermal maturity of the OM from BS samples (Ngimbang Fm.), evaluated using the Rock-Eval method, corresponds to the oil window zone (T_max_ 433–446 °C after extraction) (Fig. 6b, Supplementary Table S3). Recalculating the T_max_ parameter to the R_o_ equivalent using the formula by Jarvie *et al*.^41^ (R_o_ eq.(%) = 0.0180 × T_max_−7.16) suggests a R_o_ eq. varying between 0.63 to 0.87%. In contrast, the measured vitrinite reflectance values of the same BS clasts (R_o_ = 2.5–2.7%) are significantly higher than the estimated R_o_ using T_max_. This suggests that in the catchment area of the Lusi site the Ngimbang Fm. was exposed to temperatures significantly greater than 230 °C, based on the chemical kinetic model of vitrinite maturation proposed by Burnham and Sweeney^42^. Maceral analysis also revealed the presence of high temperature imprints, which typically take place between 400 to 1000 °C^43–45^. Palynological analyses confirm a high maturity level of the samples from the Ngimbang Fm. with spores, which were typically poorly preserved, altered cell shapes and dark brown to black colour, indicating the palynomorphs have been subjected to paleo temperatures above 250 °C^42,46^. This is consistent with chlorite and carbonate Raman microthermometry measured on the similar set of the erupted Ngimbang Fm. clasts, which indicates this formation had been exposed to conditions > 260 °C^13^.

Most sedimentary basins worldwide are characterized by relatively gradual burial, which typically leads to the coherent thermal alteration of the OM and progressive changes of the maturity-related parameters (i.e. vitrinite reflectance, T_max_, biomarker isomerisations, spore and pollen alterations). Rapid heating events induced by magmatic activity and/or hydrothermal fluid circulation may enhance the maturation of OM and unevenly modify it, leading to maturity estimate inconsistencies. For instance, comparisons of extractable organic matter (EOM) compounds in Scottish Carboniferous coals affected by contact and burial metamorphism showed that reversals in the trend of molecular-maturity parameters stem from different reaction rates of organic components in the areas subjected to rapid heating events^47^. A geochemical study of Jurassic sediments affected by Paleogene dykes also show that different maturity-related proxies show varying sensitivity to short temperature pulses^48^. Therefore, only certain proxies for OM maturation follow the expected heating rate in contact metamorphic zones, while other parameters used for basins with gradual burial do not keep pace with the thermal effects. For example, sterane and hopane maturation appears to lag behind the alteration of vitrinite macerals during short-lived temperature anomalies^49^, acquired during experimental maturation. Moreover, studies of shales affected by sill intrusions have scatter T_max_ measurements that do not follow the maturation trend estimated by vitrinite reflectance data^50,51^. Overall, multiple studies on the coal/organic matter maturation impacted by the magmatic intrusions imply that several parameters strongly affect the maturation in those specific geological settings: the lithological type of the host rock, initial maturation level of the OM prior to the intrusion, heating rate, duration, sill thickness, temperature and pressure regimes^52–55^. Therefore, the discrepancies in the maturity/temperature estimations using various maturity-related parameters mirror the short-termed high temperature anomaly occurring in the LUSI system.

Our data clearly demonstrates a complex geological setting of the southern East Java basin. Lusi is located 10 km to the NE from the active Quaternary volcanic arc. Ambient noise tomography investigations highlighted the connection between the magma chamber of the nearest volcano and the Lusi conduit at a depth of ~ 4.5 km^11^. This suggests the presence of a magmatic intrusion penetrating the organic-rich sediments of the Ngimbang Fm. and associated hydrothermal fluid migration^11^. These conclusions are supported by the gas geochemical parameters, such as presence of the mantle-derived volatiles in the gas vented at Lusi^12,14^. Furthermore, the erupted HC gases have quite different molecular and isotopic composition (δ^13^C_CH4_ up to −35.7‰) compared to the gas stored in the adjacent HC fields (δ^13^C_CH4_ range from −58.3 to −40.7‰), hence suggesting that these two systems are compartmentalised^12,16^. These lines of evidence, together with the geochemical results from the rocks clasts and oils presented here, indicate that the erupted HCs have been recently generated within the Ngimbang Fm. that is affected by a recent (Holocene?) magmatic intrusion and hydrothermal fluid migration. These rapid heating events are thus interpreted to be the reason of the discrepancies observed in the BS maturity-related parameters. Furthermore, if HC reservoirs or source rock intervals are exposed to high temperatures (>200 °C) over a geological time span, bitumen (and/or oil) is usually totally converted to gas and pyrobitumen due to secondary cracking processes^56,57^. The investigated BS samples of the Ngimbang Fm. are oversaturated with the bitumen phase (up to 43 mg HC/g rock of the extracted bitumen). Therefore, the exposure of these samples to high temperatures (>200 °C) must have taken place over a short period of time, insufficient to cause total secondary cracking of all bitumen and oil.

Our results highlight the importance of the timing effect in the maturation of the source rock. More specifically, multiple lines of evidence suggest that organic-rich sediments have been recently exposed to high temperatures (geochemically corresponding to the late gas window-overmature zone). This implies that the source rocks did not fully release their hydrocarbon potential and are essentially still prone to generate oil. Therefore, the methods to estimate thermal maturity, such as Rock-Eval and maturity-related biomarkers, will not accurately represent true paleo-temperatures in sedimentary successions affected by magmatic intrusions. Indeed, the Rock-Eval analysis and aliphatic compounds of the bitumen extracts from BS indicate that in the study area the Ngimbang Fm. was generally exposed to temperatures not higher than 120 °C (middle oil window). This would result in a thermal gradient 22–25 °C/km prior to magmatic/hydrothermal activity. A similar low gradient is also measured in the wells of the KE structure (40 km east of Lusi). In contrast, the geothermal gradient estimated at the Lusi site (42 °C/km, based on the BJP1 well^20^) implies that the Ngimbang Fm. reaches temperatures between 186–236 °C (3.8–5 km depth, 26 °C at the surface) even when applying a linear curve gradient (Fig. 2). These temperature estimates are indeed consistent with random vitrinite reflectance data and microthermometry measurements conducted on the BS clasts. The combined observations support the conclusion that a recent increase of the heat flow is localized in the southern part of the East Java sedimentary basin affected by Quaternary volcanism. The kinetic model for the BS kerogen suggests that HC generation starts at ~105 °C, and the transformation ratio of the kerogen is less than 10% at 120 °C^58^. Therefore, we cannot exclude the scenario where the HC generation processes have started prior to the migration of the magmatic/hydrothermal fluids in the study area. However, we suggest that recent magmatic activity has largely enhanced the maturation of OM and HC formation is very likely ongoing.

The distinctive difference in Hg/TOC ratios between BS and GS clasts further support the stratigraphical assessment of the clasts using biostratigraphical and geochemical methods, as well as our conclusion on the exposure of the Ngimbang Fm. to hydrothermal/magmatic activity. Elevated Hg/TOC ratios have been used as a proxy for enhanced volcanic activity in the geological record due to Hg deposition through other avenues such as clay particles^37,59^. As a result, there is now a wealth of data on Hg/TOC ratios in shales, both during periods of elevated global volcanic activity and relative global quiescence^59,60^ (Fig. 5). Compared to these data sets, BS clasts of the Ngimbang Fm. are extremely depleted in mercury, even though the study area has been located close to the volcanic arc since the Eocene. In contrast, GS samples of Miocene-Pleistocene age plot within the field of previously measured Hg/TOC ratios (Fig. 5). We suggest that preferential Hg loss occurs in the BS samples due to the volatilization and escape of Hg at high temperatures due to the recent volcanic activity. Organic-bound Hg release occurs at the temperature window of 150–400 °C, with the highest intensity at ~300 °C^61^. This provides further support for the presence of the high temperature anomaly affecting the Ngimbang Fm. at depths greater than 4 km.

Our dataset reveals also the striking maturity discrepancies between the oil present in the HC reservoirs and that erupted at Lusi. Maturity-related biomarker parameters (C_29_ sterane ββ/(ββ + αα) and 20 S/(20 S + 20 R) isomer ratios, C_27_ dia- and regular steranes ratio, Ts/Ts + Tm hopanes, moretanes/hopanes) indicate that Lusi oil films are less mature than the oil trapped in the production fields (Fig. 7c,d, Supplementary Table S1 and Fig. S1). The ratios of aromatic compounds MPI-1 and MPR (methylphenanthrene index and methylphenanthrene ratio), that are less susceptible to degradation and evaporation loss, also suggest the lowest maturity for the Lusi oil, but in contrast indicate the highest maturity for the bitumen extracts of the BS samples. Recalculated vitrinite reflectance of the BS from MPI-1 equals to 1.76 to 1.92%^29,62^. However, the reversal trend for MPI-1 may differ due to rapid heating^47^, indicating potentially higher recalculated value. Maturity estimates based on phenanthrene ratios thus result in high uncertainty.

**Figure 8:**
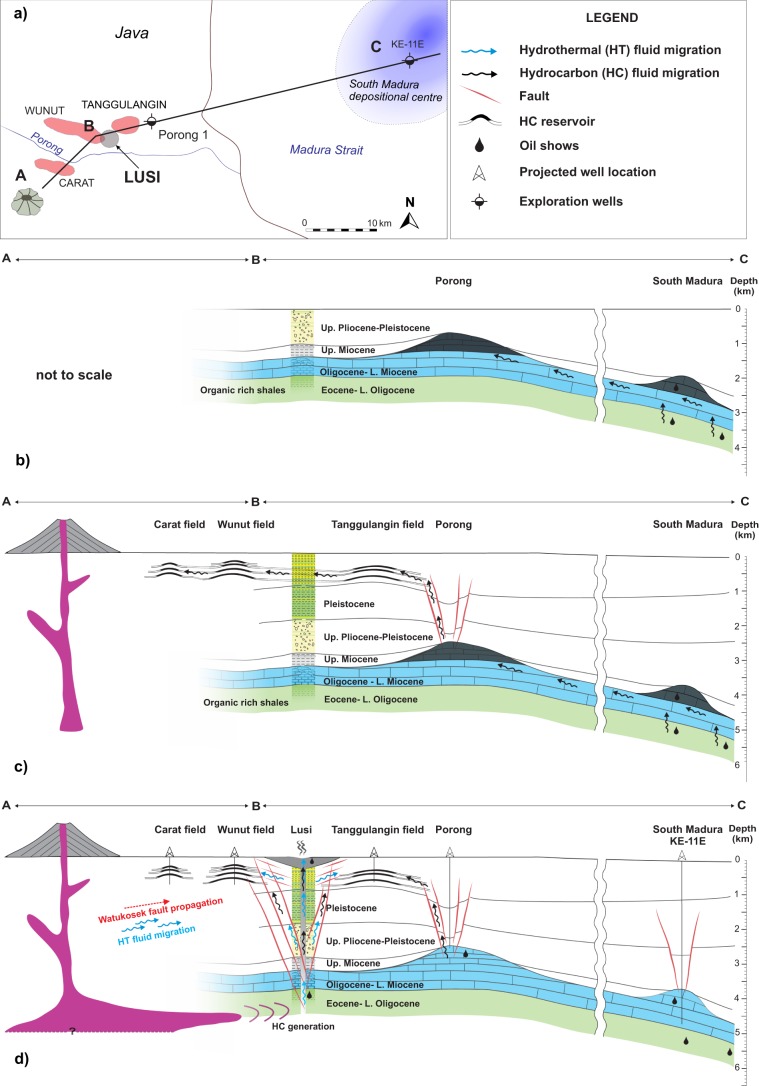
Conceptual geological model depicting the development of the petroleum system in the study area: (**a**) location of the profile ABC, crossing HC fields, Lusi eruption site, Porong paleovent and South Madura depositional center; (**b**) HC generation in the subsided South Madura region (on the east) and migration to the Porong reefal carbonates during Late Miocene-Pliocene; (**c**) collapse of the Porong trap in Pleistocene or recent, followed by the secondary migration of the HCs to the shallow Pleistocene reservoirs (Tanggulangin, Wunut, Carat fields); (**d**) Holocene magmatic intrusion penetrates organic-rich deposits (>4 km depth), triggering HC and CO_2_ generation below Lusi and overpressure buildup. Reactivation of the Watukosek fault system was followed by the occurrence of the Lusi piercement and surface migration of the brecciated sediments, water, hydrocarbons and mantle-derived fluids.

Our multidisciplinary study provides insights into understanding the Lusi plumbing system and the HC migration mechanisms at regional scale. Specifically, the maturity differences imply that: a) HC accumulations of Wunut and Tanggulangin fields are not the source of the large volume of oil erupted from the Lusi system; and b) the oils from the HC fields and Lusi were likely generated at different depositional areas of the Ngimbang Fm. and therefore most probably had different migration pathways. Integrating all the geological and geochemical observations, we propose a conceptual model of the hydrocarbon migration in the southern part of the NE Java Basin (Fig. 8a–d):

- Late Miocene-Pliocene HC generation initiated within the organic-rich shales of the Ngimbang Fm. deposited in the deepest parts of the East Java sedimentary basin where the South Madura depositional centre resides (Fig. 8b). Gravity anomalies and well data show that in this part of the basin (located ~40 km to the east of Lusi) the sedimentary cover is thicker and hence Ngimbang Fm. is buried at least 1 km deeper^63,64^. Therefore, deep burial induced HC generation most likely occurred in the South Madura region.
- HC migration to the carbonates of the Kujung Fm., forming the accumulation within the Porong reefal structure, located 7 km to the north-east of Lusi^64^. The Porong-1 exploration well penetrated the top part of this potential reservoir revealing the presence of oil shows^26^ (Fig. 8b).
- Late Pleistocene-Holocene witnessed the triggering and collapse of the Porong paleo-vent. The breach of the seal led to secondary HC migration from the Porong trap to the shallow clastic units of the Pucangan Fm.^64^, forming the shallow accumulations of the Tanggulangin, Wunut and Carat fields (Fig. 8c).
- Holocene migration of magmatic fluids within the organic-rich Ngimbang Fm. triggered thermo-metamorphic reactions, greatly enhancing the generation of the HCs, CO_2_ and leading to overpressure build-up. The recent reactivation of the Watukosek fault system^65^ facilitated the trigger of the Lusi system and the release of HC at the surface (Fig. 8d).

The Lusi eruption represents an excellent opportunity to investigate the impact of the recent activity of volcanic systems on petroleum sedimentary basins and enhanced HC generation. The study highlights the relevance of time factor for source rock maturation, and the importance of specific geochemical and microscopy methods (i.e. palynology, organic petrology, and chlorite microthermometry) to estimate the temperatures in sedimentary basins affected by volcanic activity.

## Methods

### Bulk analyses (C_org_, Rock-Eval)

Bulk rock analyses were performed at the Federal Institute for Geosciences and Natural Resources (BGR, Hannover, 12 samples), and at the Applied Petroleum Technology (APT, Oslo, 18 samples) laboratories. Both laboratories used the same Rock-Eval 6 analyzer standard procedures.

Aliquots of the dried (at 40 °C for 48 h) clasts samples were crushed and ground (grain size <200 μm) using a mortar grinder mill. Rock-Eval pyrolysis was performed on a Rock-Eval 6 analyzer using a standard program^66,67^: start isothermal with 300 °C for 3 min, then applying a heating rate of 25 °C/min up to 650 °C. Initial sample weights were between 10 and 200 mg depending on the expected S_2_ yield to prevent oversaturation of the FID for highly productive samples. Hydrocarbons, released isothermally at 300 °C are presented as S_1_ and between 300 and 650 °C as S_2_ yields. Precision of the hydrocarbon determination was better than 5%. T_max_ values represent the maxima of the S_2_ peak and correspond to the evaluation of the thermal maturity of the organic matter. For better estimation of the residual generative potential of the kerogen Rock-Eval analyses were as well performed on the extracted samples.

Organic carbon was measured with Rock-Eval 6 analyzer at APT labs. At BGR labs. organic carbon was measured with LECO CS-230 (Leco Instrumente, Germany). The samples were primarily decalcified (acidification with 10% hydrochloric acid; HCl at 80 °C) and dried (50 °C for 18 h). About 180 mg of each sample was burned in a high-frequency induction furnace in an oxygen atmosphere by use of the absorption signal at the IR detector. The instrument was calibrated using commercially available standards (LECO). Reproducibility of the measurements (organic and carbonate carbon content) was ± 0.02%.

### Extraction and biomarker analysis

Analyses of mid- to high-molecular-weight hydrocarbons were performed at the BGR labs. Organic compounds were extracted from the grounded clast samples (3–10 g) 3 times using a 10–30 ml mixture of dichloromethane-methanol (DCM:MeOH, 8:2; volume:volume) for 15 min in an ultrasonic bath. All triplicate extracts were combined. The oil films from the Lusi crater mud samples (40–120 g) were extracted with 20 ml DCM by shaking for 5 min and subsequent separation of the organic phase. All extracts were dried under a nitrogen stream at 40 °C and weighed. All extracts were transferred to a chromatographic column filled with activated silica gel (240 °C for 12 h) and fractionated into an aliphatic and aromatic fraction using isohexane and DCM respectively. All fractions were dried under a nitrogen stream and weighed.

The oil-field samples were stabilised at 40 °C for 18 h and subjected to a fractionation procedure. Prior to this, asphaltenes in the oils were precipitated by adding 2 mL DCM and 60 mL petroleum ether to (at maximum) 100 mg of oil (reaction time 12 h). Subsequently, the solutions were centrifuged at 3000 rpm for 15 min. The supernatant solution containing maltenes and resins was collected and the solvent removed through evaporation in a nitrogen atmosphere at 40 °C. Parallel preparation and asphaltene precipitation of a sample of known composition (Norwegian Geochemical Standard NSO-1 oil) assured reproducibility control of the method. The residual maltenes and resins (up to 100 mg) were separated into aliphatic and aromatic fractions as well as into hetero-compounds (NSO-compounds) on silica gel (activated at 240 °C for 12 h) by mid-pressure liquid chromatography (BESTA-Technik für Chromatographie GmbH, using a sequence of organic solvents of different polarity (iso-hexane, iso-hexane/DCM (mix 2:1; v-v), DCM/MeOH (mix 2:1; v-v)).

The distribution of compounds contained in the aliphatic fractions was determined with an Agilent 7890 gas chromatograph (GC) equipped with a 50 m Ultra 1 column (Agilent; 0.2 mm inner diameter; 0.11 µm film thickness) and connected to a flame ionization detector (FID). Individual biomarkers were analysed after gas chromatographic separation (Agilent 7890) with a mass spectrometer system (MS; Agilent QQQ 7000). Measurements of aliphatic fractions were carried out as multiple-reaction*-*monitoring using parent-daughter-scans. The aromatic fractions were analysed in the full-scan mode (scanning from *m/z* 50 to 700). Compounds were identified by comparison of mass spectra and retention times with own and published data. Sterane and hopane biomarkers ratios were calculated from individual peak areas.

### Organic petrography

The preparation of polished particulate blocks was conducted by LAOP, Tübingen, Germany following the German Standard Methods DIN22020-2:1998-08^68^ and the guidelines published in Taylor *et al*.^69^. Random huminite and vitrinite reflectance measurements were carried out in accordance to German National Standard DIN22020-5:2005-02^70^, under non-polarized light, at magnification of 500× and room temperature of 23 °C ± 1 °C using a Leica DMRX incident-light microscope equipped with a MPV Compact 2 microphotometer photomultiplier tube (PMT), halogen lamp (12 V, 100 W), HBO® Lamp (103 W/2, 12 V), and Leica Oil P 50×/0.85 oil immersion objective. Leica Type F immersion oil ne = 1.518 (23 °C). Up to twenty five random reflectance measurements were performed on the rock samples using Leica MPV Measure software. The filters used for analysis in fluorescence mode were Leica excitation filter BP 355–425, dichroic mirror RKP 455, and barrier filter LP 460. Photomicrographs were captured under incident white and blue light excitation using a Leica digital fluorescence camera DC 300 F at format of 1.300 × 1030 pixels and were stored using imaging software Image Access Premium 09. The maceral nomenclature applied in this paper follows ICCP System 1994, as it is adopted by the International Committee for Coal and Organic Petrology^71–73^.

### Palynological preparations

Samples preparation and analyses were carried out at the Department of Geosciences, University of Oslo. About 10 g of sediment was crushed and treated with acids to remove the rock-mineral components according to palynological standard protocols adopted from Traverse^74^. 10% HCl at room temperature to dissolve the carbonate fraction. To dissolve the silicates, the samples were treated with hot concentrated HF (65 °C) in a water bath for two days. The organic residue was washed and rinsed sieved with a 250 µm and a 15 µm mesh. Slides were mounted using epoxy resin (Entellan) as a mounting medium.

The organic residue of the highly thermally altered BS samples was treated in a series of experiments in test tubes with 3 different bleaching agents to extract and lighten recognizable palynomorphs or any other particulate present in the dark-coloured organic matter fraction. The tested bleaching reagents included: a) NaOCl solution (5%), b) concentrated nitric acid HNO_3_ and c) Schulze’s solution (saturated K_2_ClO_3_+ concentrated HNO_3_). On selected samples an additional treatment with NaOCl solution (5%) was applied at 40 °C for 10 h. Among the applied bleaching methods, only the samples treated with Schulze’s solution released small organic particles.

Photographs were taken with a Zeiss Imager and Zeiss digital camera at 400x magnification. The organic residues are stored at the Department of Geosciences, University of Oslo, Norway.

### Mercury analysis

Mercury analyses were conducted at the University of Oxford using a Lumex RA-915 Portable Mercury Analyzer with an attached PYRO-915 pyrolyzer^75^. Analytical procedures followed established in-house protocols^60,76^ and calibrated using the NIMT/UOE/FM/001 peat standard with a known Hg concentration of 169 ± 7 ppb. Between 50 to 100 mg of powdered sample was weighed before being inserted into the pyrolyzer. The samples were heated to >700 °C and left for up to 120 seconds to allow full volatilization of the Hg present. The machine was recalibrated every ten samples to negate any influence of drift in the sensor. As such, individual sample analytical errors are only ±5%, although samples with Hg concentrations <5 ppb are subject to greater errors due to background noise affecting peak integration.

## Supplementary information

Supplementary material

## Data Availability

All data generated or analysed during this study are included in this published article and its Supplementary Information files.
