# Supplementary material for: Recent magmatism drives hydrocarbon generation in north-east Java, Indonesia
Source: Sci Rep. 2020 Feb 4;10:1786. doi: 10.1038/s41598-020-58567-6 (PMC7000390; doi:10.1038/s41598-020-58567-6)
Supplement: Supplementary file 1 — Supplementary material [file 41598_2020_58567_MOESM1_ESM.pdf]

Supplementary Material for the paper:

"Recent magmatism drives hydrocarbon generation in north-east Java, Indonesia".

Alexandra Zaputlyaeva (1), Adriano Mazzini (1), Martin Blumenberg (2), Georg Scheeder (2), Wolfram Michael Kürschner (3), Jolanta Kus (2), Morgan Thomas Jones (1), Joost Frieling (4)

(1) Centre for Earth Evolution and Dynamics (CEED), University of Oslo, Norway
(2) Federal Institute for Geosciences and Natural Resources (BGR), Hannover, Germany
(3) Department of Geosciences, University of Oslo, Norway
(4) Department of Earth Sciences, Utrecht University, Netherlands

Supplementary Table S1. Main biomarker parameters, methylphenanthrene index (MPI-1=1.5*(2-MP+3-MP)/(P+1-MP+9-MP) and methylphenantrene ratio (MPR=2-MP/1-MP), and dibenzothiophene/phenanthrene ratio (DBT/Phen) of the studied bitumen extracts of the grey shale (GS), black shale (BS), and oil (O) samples. Oil was sampled from the Lusi crater and as well from Wunut and Tanggulangin oil fields, located close to the Lusi.

Sample ID	Sample type	Sampling location	Dia/Dia+Reg C_{27}	20S/20S+20R dia C_{27}	moretanes/ hopanes	Ts/Ts+Tm	homohopane index	C_{32} hopane (22S/22S+22R)	C_{29} 20S(20S+20R)	C_{29} ββ/(ββ+αα)	CPI	Pr/Ph	Ol/(Ol+Hop)	2-M/ 2-M+Hopan.	3-M/ 3-M+Hopan.	MPI-1	MPR	DBT/Phen
JV17-01-39	GS	Lusi crater	0.02	0.47	0.17	0.25	-	0.31	0.02	0.10	1.61	2.46	0.32	0.025	0.026	0.56	1.17	0.12
JV17-01-40	GS	Lusi crater	0.01	0.41	0.27	0.10	-	0.31	0.01	0.16	1.43	0.50	0.47	0.026	0.023	0.41	1.06	0.10
JV16-29-01	BS	Lusi crater	0.40	0.58	0.05	0.59	3.33	0.56	0.45	0.50	1.02	3.55	0.17	0.051	0.034	0.79	1.39	0.59
JV16-29-04	BS	Lusi crater	0.40	0.59	0.06	0.57	3.13	0.56	0.45	0.51	1.02	2.94	0.20	0.053	0.039	0.65	1.22	0.80
JV17-01-01	BS	Lusi crater	0.39	0.58	0.05	0.61	2.19	0.57	0.45	0.50	1.04	3.69	0.23	0.052	0.034	0.63	1.03	0.47
JV17-01-10	BS	Lusi crater	0.36	0.60	0.06	0.59	2.98	0.57	0.42	0.50	1.02	2.56	0.20	0.060	0.034	0.79	1.57	0.55
JV17-01-15	BS	Lusi crater	0.37	0.57	0.05	0.58	3.44	0.58	0.41	0.48	1.03	3.12	0.16	0.054	0.033	0.90	1.33	0.80
JV17-01-37	BS	Lusi crater	0.41	0.61	0.05	0.59	3.19	0.56	0.44	0.51	1.03	3.48	0.17	0.053	0.035	0.80	1.49	0.72
JV17-40	O	Wunut f.	0.44	0.62	0.05	0.62	3.63	0.56	0.50	0.54	1.03	4.13	0.18	0.058	0.031	0.73	1.22	0.31
JV17-47	O	Tang f.	0.46	0.62	0.05	0.62	3.39	0.57	0.51	0.55	1.00	4.26	0.17	0.057	0.033	0.76	1.22	0.55
JV17-48	O	Tang. f.	0.45	0.60	0.05	0.64	3.62	0.58	0.51	0.55	0.94	4.34	0.20	0.056	0.031	0.78	1.19	0.45
JV17-50	O	Wunut f.	0.49	0.60	0.05	0.66	3.47	0.57	0.53	0.56	1.06	4.31	0.24	0.059	0.031	0.82	1.22	0.48
JV15-A	O	Lusi crater	0.30	0.60	0.07	0.56	2.96	0.60	0.29	0.40	1.02	3.93	0.20	0.054	0.030	-	-	-
JV15-B	O	Lusi crater	0.30	0.57	0.07	0.56	3.18	0.58	0.28	0.41	1.02	3.93	0.19	0.057	0.034	-	-	-
JV17-A	O	Lusi crater	0.25	0.56	0.07	0.56	3.10	0.55	0.20	0.29	1.03	2.58	0.18	0.054	0.031	0.62	1.18	0.38
JV17-B	O	Lusi crater	0.24	0.58	0.08	0.56	3.11	0.54	0.19	0.27	1.03	2.92	0.18	0.053	0.033	0.55	1.18	0.54

Supplementary Table S2 Results of the palynological analyses of the rock clasts. Highly thermally-altered samples were bleached using NaOCl solution (5%), concentrated nitric acid (HNO_3), and Schulze's solution (saturated K_2ClO_3 + concentrated HNO_3). Original – preparations without bleaching procedure; bl. – bleached; TOC – Total Organic Carbon; TAS – Thermally Alteration Scale, developed by Batten [33]; VR – Vitrinite Reflectance; AOM – Amorphous organic matter. Yellow, orange and grey colours highlight presence of 3 sample groups with various maturation, based on the spore and pollen colours.

Sample ID	Preparation type	Lithology	TOC, wt.%	Palynofacies	TAS (according to Batten, 1996)	Palynology	Stratigraphic age
**JV14B-11-01**	original	light grey shale	0.34	light brown plant debris (plenty cuticle and vitrinite), light brown AOM	1-2 (<50 °C) VR 0.2-0.3 %	*Lingulodinium machaerophorum*, *Tasmanites*, smooth walled spores, diverse angiosperm pollen, mangrove palm pollen (*Nypa*)	Pleistocene?
**JV14B-11-02**	original	grey shale	0.35	light brown - brown plant debris (plenty cuticle and vitrinite), light brown AOM	1-2 (<50 °C) VR 0.2-0.3 %	no	no age assignment possible
**JV14B-11-02**	bl. Schultze's S.	grey shale	0.35	light brown - light brownish plant debris (plenty cuticle and vitrinite), light brown AOM		no	no age assignment possible
**JV14B-11-11**	original	grey shale	1.99	light brown plant debris (plenty cuticle and vitrinite), light brown AOM	1-2 (<50 °C) VR 0.2-0.3 %	no	no age assignment possible
**JV17-01-39**	original	grey marl, immature,	0.83		1-2 (<50 °C) VR 0.2-0.3 %		
**JV17-01-40**	original	grey marl, immature,	0.95	light brown plant debris (cuticle, vitirinite), foraminiferal linings, AOM	1-2 (<50 °C) VR 0.2-0.3 %	*Spiniferites, Lingulodinium, Selenopemphix armata?, Diphyes (or Dapsilidinium?)*	Late Pliocene-Pleistocene
**JV14B-02-02**	original	dark grey shale	0.5	light brown plant debris (plenty cuticle and vitrinite), light brown AOM	6 (170-180 °C) VR 1.5-2%	*Lingulodinium machaerophorum, Operculodinium centrocarpum and O. piaseckii, Polykrikos schwartzii* (or *kofoidii*), *Spiniferites, Stelladinium* (*Lejeunecysta*), spores, *Ruellia* type pollen?	Middle - Late Miocene

**JV15-06-08**	original	dark grey shale	1.27	dark brownish to black plant debris (vitrinite, cuticles), degraded dark AOM	6 (170-180 °C) VR 1.5-2%	lacking identifiable palynomorphs	Pleistocene or Miocene
	bl. Schulze's S.			dark brownish to black plant debris (vitrinite, cuticles)		*Spiniferites bentorii*	
**JV17-01-02**	original	grey brecia with volcanoclastics	0.48		5-6 (150-180 °C) VR 1.5-2%		no age assignment possible
**JV17-01-08**	original	grey shale	0.82	dark brownish AOM, plant debris, foraminiferal linings	5-6 (150-180 °C) VR 1.5-2%	*Spiniferites*, *Operculodinium*, *Palambages?*, striate angiosperm pollen	Miocene?
**JV17-01-34**	original	grey shale	0.55	dark brownish to black plant debris (vitrinite, cuticles)	5-6 (150-180 °C) VR 1.5-2%	no	no age assignment possible
**JV15-06-04**	original	coal with leaf imprints	66.4	dark brownish and black AOM, dropplets of resin	7 (>250 °C) VR 4%	lacking identifiable palynomorphs	no age assignment possible
	bl. Schulze's S.			dark brownish and black AOM, dropplets of resin			
**JV15-06-05**	original	black shale	1.59	dark brownish to black plant debris (vitrinite, cuticles), degraded dark AOM	7 (>250 °C) VR 4%	lacking identifiable palynomorphs	no age assignment possible
	bl. Schulze's S.			dark brownish to black plant debris, dominating vitrinite			
**JV16-29-01**	original	black shale	9.21	black opaque plant debris, degraded dark grey AOM	7 (>250 °C) VR 4%	lacking identifiable palynomorphs	no age assignment possible
	bleached NaOCl			no visual difference from the original			
	bl. NaOCl+temp						
	bl. HNO3						
	bl. Schulze's S.			yellow AOM, plant debris, vitrinite			
**JV16-29-04**	original	black shale	14.6	black opaque or dark brownish plant debris (vitirinite, cuticle)	7 (>250 °C) VR 4%	lacking identifiable palynomorphs	no age assignment possible
	bleached NaOCl			no visual difference from the original			
	bl. NaOCl+temp						
	bl. HNO3						
	bl. Schulze's S.			brown plant debris (vitrinite, cuticle) *Botryococcus*		angiosperm pollen, spores	

**JV17-01-01**	original	dark grey shale	2.6	dark brown plant debris (vitrinite, cuticles), no AOM	5-6 (150-180 °C) VR 1.5-2%	spores (smooth, spinous, faveolate)	no age assignment possible
	bl. Schulze's S.			light brown plant debris, mainly vitrinite and cuticles, no AOM			
**JV17-01-10**	original	black shale	6.94	black plant debris (vitrinite, cuticle) and degraded AOM	7 (>250 °C) VR 4%	lacking identifiable palynomorphs	no age assignment possible
	bl. Schulze's S.			black plant debris (vitrinite, cuticle) and degraded AOM			
**JV17-01-15**	original	black shale	8.61	dark brownish, black opaque plant debris (vitrinite) and AOM	7 (>250 °C) VR 4%	lacking identifiable palynomorphs	no age assignment possible
	bl. NaOCl			no visual difference from the original			
	bl. NaOCl+temp						
	bl. HNO3						
	bl. Schulze's S.			dark brownish to black plant debris (vitrinite, cuticles), *Botryococcus*		algae remains?, mangrove palm pollen (*Nypa*),	
**JV17-01-37**	original	black shale	4.76	dark brownish to black plant debris (vitrinite, cuticles)	7 (>250 °C) VR 4%	lacking identifiable palynomorphs	no age assignment possible
	bl. Schulze's S.			dark brownish to black plant debris (vitrinite, cuticles)			
**JV15-06-04**	original	coal with leaf imprints	66.4	dark brownish and black AOM, dropplets of resin	7 (>250 °C) VR 4%	lacking identifiable palynomorphs	no age assignment possible
	bl. Schulze's S.			dark brownish and black AOM, dropplets of resin			
**JV15-06-05**	original	black shale	1.59	dark brownish to black plant debris (vitrinite, cuticles), degraded dark AOM	7 (>250 °C) VR 4%	lacking identifiable palynomorphs	no age assignment possible
	bl. Schulze's S.			dark brownish to black plant debris, dominating vitrinite			

Supplementary Table S3. Results of the pyrolysis analysis, using Rock-Eval method, organic carbon content (C_{org}), measured by LECO, vitrinite reflectance (R_o), bitumen extraction yield, and mercury content (Hg). Analyses using Rock-Eval and Leco instruments were performed twice: before and after extraction. BS- black shale, GC-grey shale. Suggested age assignment is based on combined palynostratigraphical and geochemical methods.

Sample ID	Group	Age	Before extraction							After Extraction					Ro, %	Bitum. extr-n yield (mg/g)	Lab	Hg (ppb)	Hg/ TOC
			S_1, (mg/g)	S_2, (mg/g)	S_3 (mg/g)	T_{max} (°C)	PI	C_{org}, (%)	C_{carb}, (%)	S_1, (mg/g)	S_2, (mg/g)	S_3 (mg/g)	T_{max} (°C)	C_{org}, (%)					
**JV14B-11-01**	GS	Plio.-Pleistocene	0.01	0.10	1.9	444	0.09	0.3									APT	14.0	41.2
**JV14B-11-03**	GS		0.04	0.04	1.7		0.50	0.4									APT	7.8	20.0
**JV14B-11-04**	GS		0.01	0.17	1.8	438	0.06	1.0									APT	47.0	49.0
**JV14B-11-05**	GS		0.01	0.17	3.3	441	0.06	0.5									APT	23.5	43.5
**JV14B-11-06**	GS		0.00	0.23	2.3	419	0.00	0.6									APT	13.0	21.3
**JV14B-11-09**	GS		0.03	0.23	3.2	437	0.12	0.9									APT	25.5	29.0
**JV14B-11-10**	GS		0.03	0.05	2.7		0.38	0.6									APT		
**JV14B-11-11**	GS		0.02	0.37	1.4	426	0.05	2.0									APT	24.5	12.3
**JV14B-02-01**	GS		0.01	0.10	1.4		0.09	0.5									APT		
**JV15-06-08**	GS		0.04	0.10	0.5		0.29	1.3									APT		
**JV15-06-09**	GS		0.11	0.18	0.8		0.38	1.2									APT		
**JV15-06-10**	GS		0.07	0.25	0.6		0.22	1.1									APT		
**JV15-GS**	GS		0.01	0.14		442	0.07	0.3									BGR	4.7	18.8
**JV-17-01-39**	GS		0.21	1.23	3.1	437	0.15	0.8	1.3	0.1	0.5	2.5	442	0.8		0.3	BGR	16.0	19.2
**JV-17-01-40**	GS	Plio.-Pleistocene	0.13	1.11	1.3	423	0.10	1.0	0.6	0.1	0.5	0.8	423	1.0	0.45	0.3	BGR	20.0	21.0
**JV14B-02-02**	GS	Miocene	0.00	0.11	1.9		0.00	0.5									APT		
**JV17-01-02**	GS	Miocene?	0.09	0.20	0.8		0.3	0.5	0.5								BGR	1.9	4.0
**JV17-01-08**	GS	Miocene?	0.31	0.63	0.1		0.3	0.8	0.0								BGR	2.9	3.5
**JV15-06-02**	BS		0.09	2.08	0.5	445	0.04	3.4									APT	1.0	0.3
**JV15-06-03**	BS		0.47	3.12	1.1	441	0.13	14.1									APT	1.6	0.1
**JV15-06-05**	BS		0.08	0.16	0.5		0.33	1.6									APT	1.0	0.6
**JV15-06-06**	BS		0.38	7.79	0.5	442	0.05	8.3									APT	1.0	0.1
**JV15-06-07**	BS		0.35	3.85	0.6	441	0.08	5.9									APT	5.1	0.9
**JV15-BS**	BS	Eocene-	0.85	7.89		444	0.10	12.0									BGR	2.4	0.2
**JV-16-29-01**	BS	Oligocene	10.16	21.24	0.2	443	0.32	9.2	0.3	0.3	8.6	0.3	445	7.8		17.4	BGR	1.0	0.1
**JV-16-29-04**	BS		19.80	27.90	0.1	442	0.42	14.6	0.3	0.5	8.9	0.1	446	11.7	2.69	43.4	BGR	8.5	0.6
**JV-17-01-01**	BS		0.89	1.69	0.1	426	0.34	2.6	0.2	0.1	0.4	0.2		2.5	2.47	1.6	BGR	1.0	0.4
**JV-17-01-10**	BS		7.00	8.90	0.1	432	0.44	6.9	0.3	0.1	2.4	0.2	438	4.5		18.6	BGR	8.3	1.2
**JV-17-01-15**	BS		15.52	22.33	0.2	440	0.41	8.6	0.4	0.2	7.2	0.2	443	6.0		40.1	BGR	1.9	0.2
**JV-17-01-37**	BS		7.89	12.70	0.2	441	0.38	4.8	0.8	0.1	2.3	0.3	433	2.8		26.7	BGR	1.0	0.2

Supplementary Figure S1. Reconstructed chromatograms of the Gas Chromatography – Mass Spectrometry (GC-MS) traces m/z 191 and m/z 217 for the bitumen extracts of the black shales (BS) and grey shales (GS), oils from the HC fields and Lusi oil films.

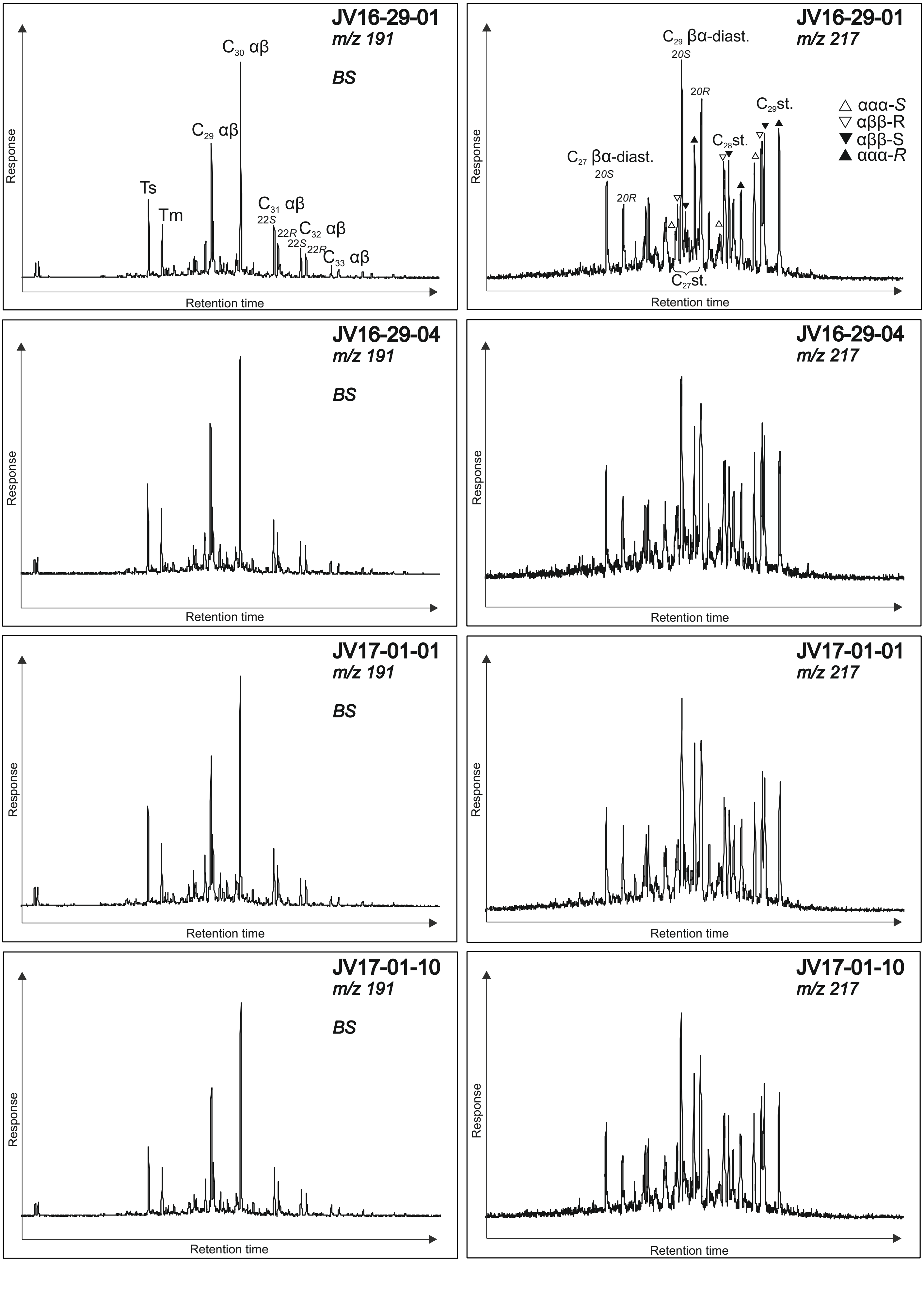

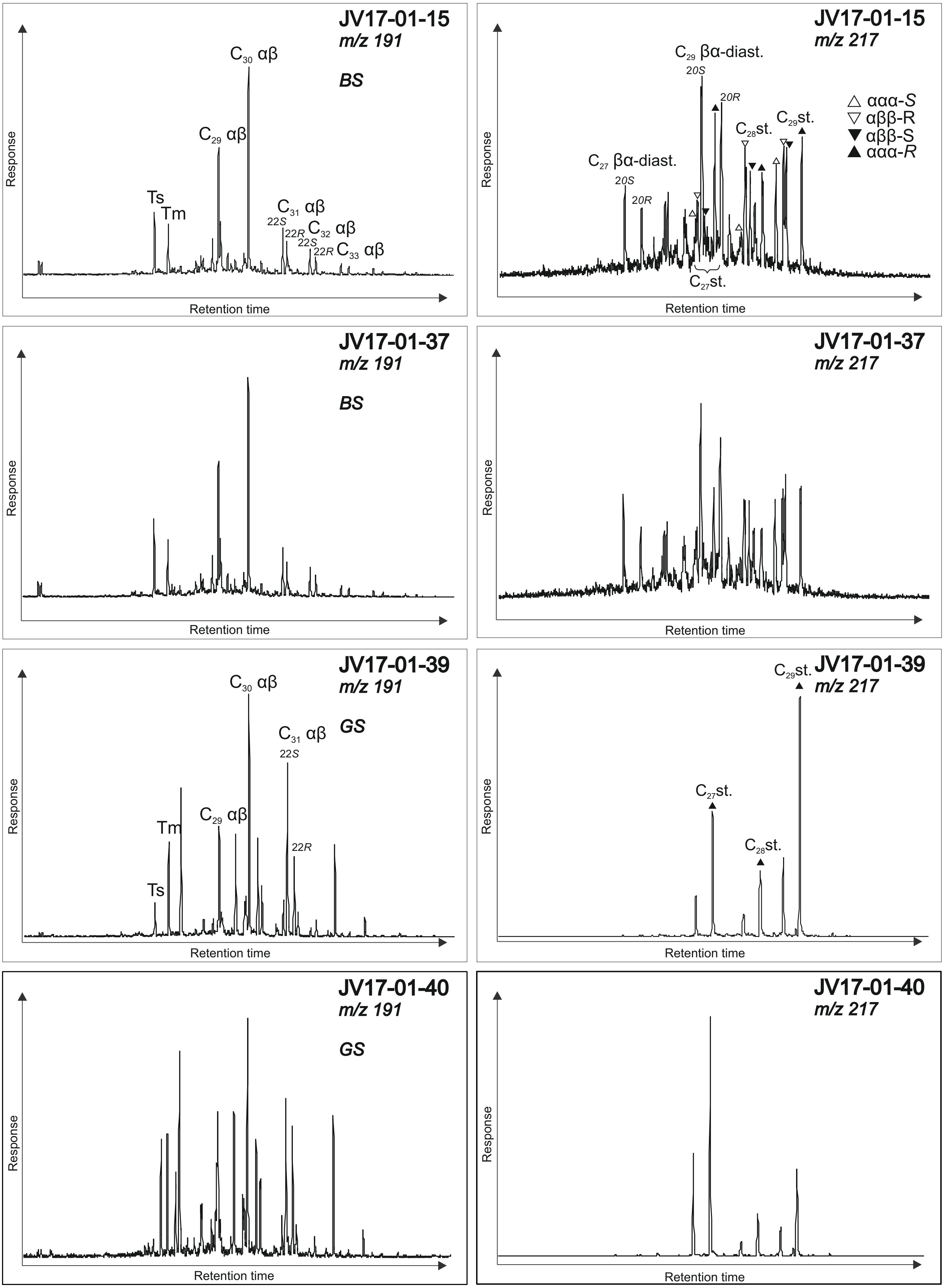
JV17-01-15
m/z 191
BS
C_{30} αβ
C_{29} αβ
Ts
Tm
C_{31} αβ
22S
22R
C_{32} αβ
22S
22R
C_{33} αβ
Response
Retention time
JV17-01-15
m/z 217
C_{29} βα-diast.
20S
20R
C_{27} βα-diast.
20S
20R
C_{28}st.
C_{29}st.
C_{27}st.
△ ααα-S
▽ αββ-R
▼ αββ-S
▲ ααα-R
Response
Retention time
JV17-01-37
m/z 191
BS
Response
Retention time
JV17-01-37
m/z 217
Response
Retention time
JV17-01-39
m/z 191
GS
C_{30} αβ
C_{31} αβ
22S
22R
C_{29} αβ
Tm
Ts
Response
Retention time
JV17-01-39
m/z 217
C_{29}st.
C_{27}st.
C_{28}st.
Response
Retention time
JV17-01-40
m/z 191
GS
Response
Retention time
JV17-01-40
m/z 217
Response
Retention time

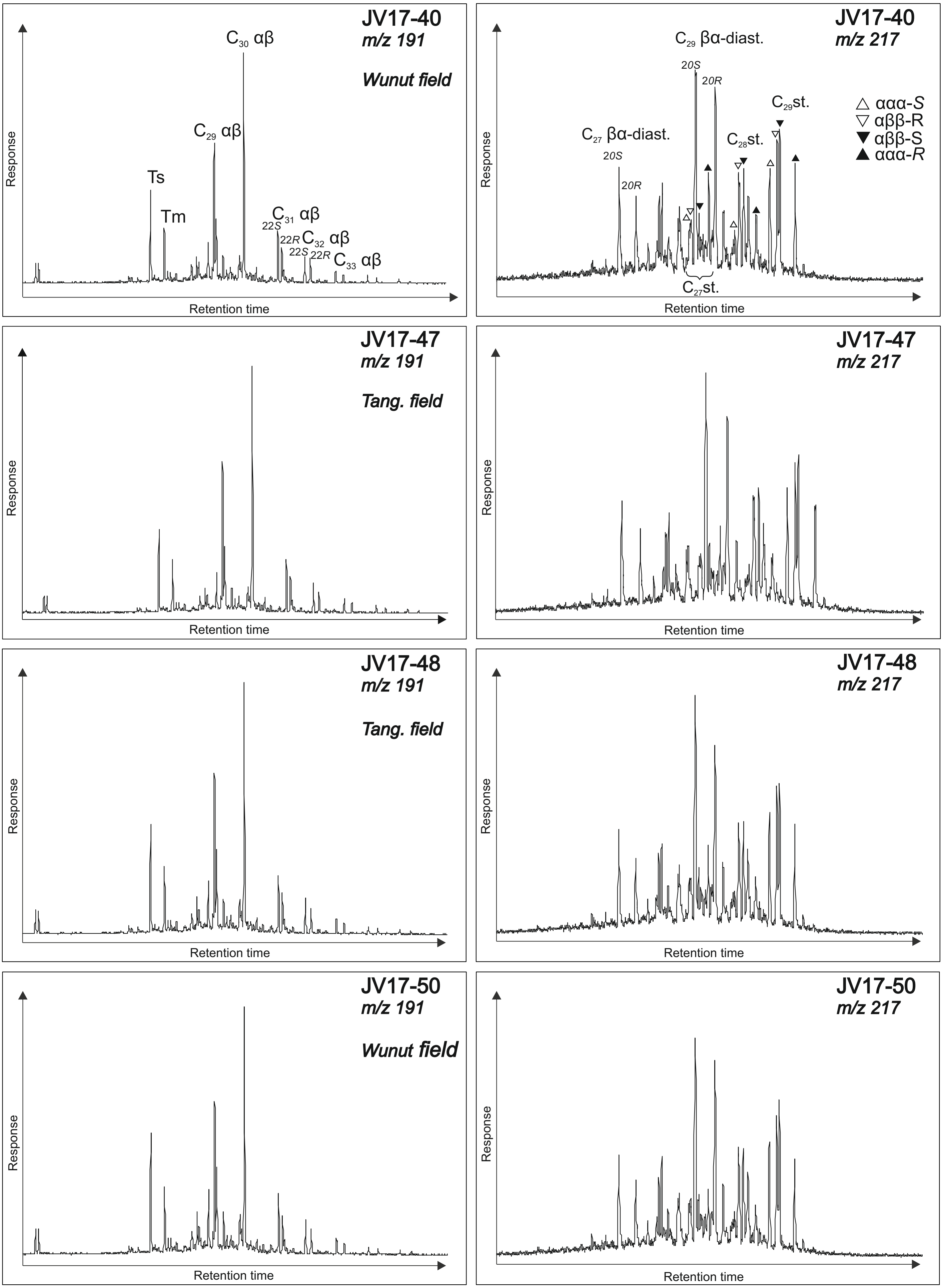
JV17-40
m/z 191
Wunut field
C30 αβ
C29 αβ
Ts
Tm
22S
C31 αβ
22R
C32 αβ
22S
22R
C33 αβ
Response
Retention time
JV17-40
m/z 217
C29 βα-diast.
20S
20R
C29st.
△ ααα-S
▽ αββ-R
▼ αββ-S
▲ ααα-R
C27 βα-diast.
20S
20R
C28st.
C27st.
Response
Retention time
JV17-47
m/z 191
Tang. field
Response
Retention time
JV17-47
m/z 217
Response
Retention time
JV17-48
m/z 191
Tang. field
Response
Retention time
JV17-48
m/z 217
Response
Retention time
JV17-50
m/z 191
Wunut field
Response
Retention time
JV17-50
m/z 217
Response
Retention time

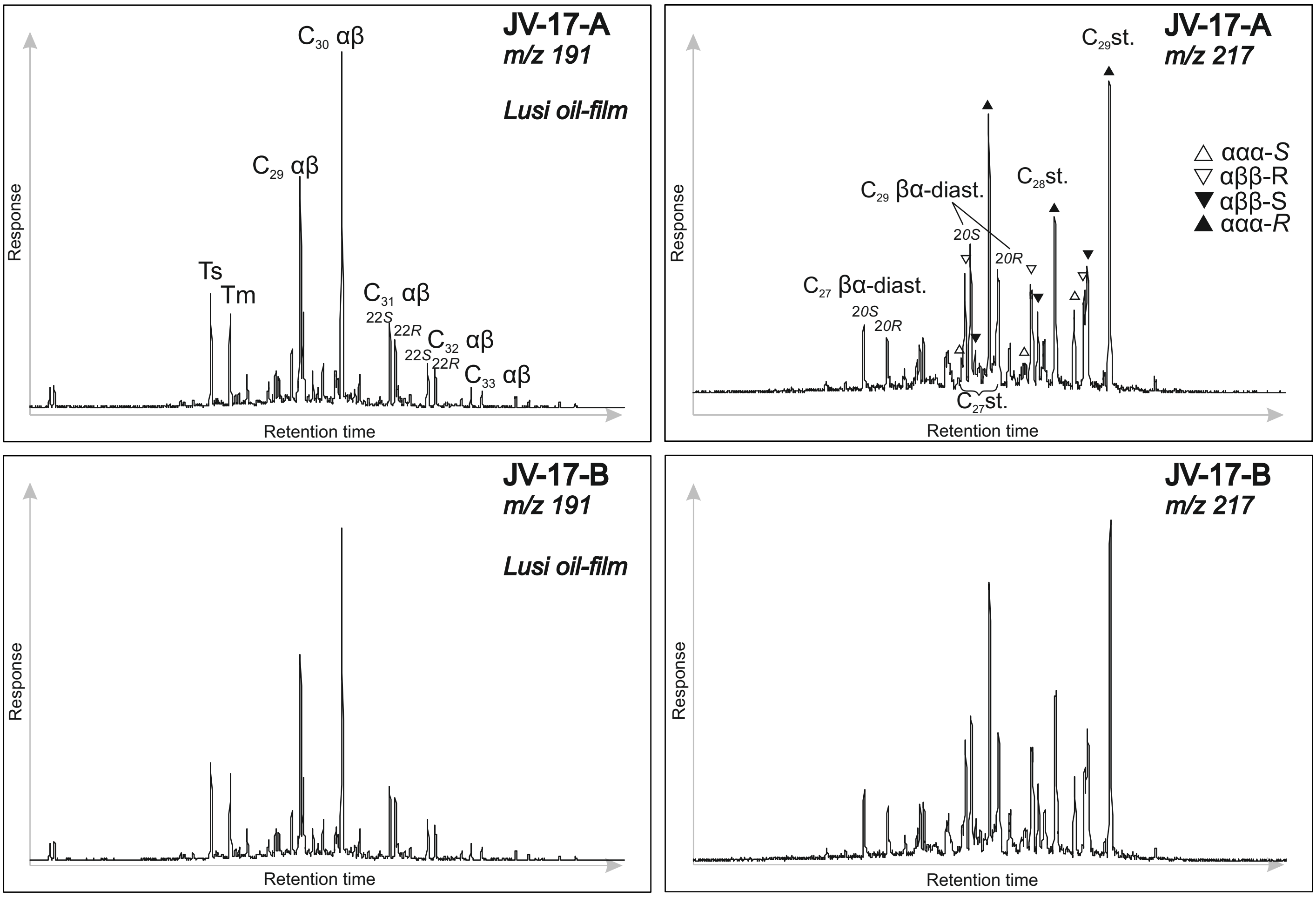
JV-17-A
m/z 191
Lusi oil-film
C_{30} αβ
C_{29} αβ
Ts
Tm
C_{31} αβ
22S
22R
C_{32} αβ
22S
22R
C_{33} αβ
Response
Retention time
JV-17-A
m/z 217
C_{29}st.
△ ααα-S
▽ αββ-R
▼ αββ-S
▲ ααα-R
C_{28}st.
C_{29} βα-diast.
20S
20R
C_{27} βα-diast.
20S
20R
C_{27}st.
Response
Retention time
JV-17-B
m/z 191
Lusi oil-film
Response
Retention time
JV-17-B
m/z 217
Response
Retention time
